# Comparative Studies on the Structure and Biological Activities of Two New Polysaccharides from *Tricholoma sinoportentosum* (TS-P) and *Termitomyces albuminosus* (TA-P)

**DOI:** 10.3390/polym15092227

**Published:** 2023-05-08

**Authors:** Xi Chen, Tong Yang, Qinghua Huang, Biao Li, Xiang Ding, Yiling Hou

**Affiliations:** 1 College of Environmental Science and Engineering, China West Normal University, Nanchong 637009, China; 2 Key Laboratory of Southwest Wildlife Resource Conservation, Ministry of Education, College of Life Sciences, China West Normal University, Nanchong 637009, China; 3 Xichong Xinghe Biotechnology Co., Ltd., Xichong 637299, China; 4 Academy of Agricultural Sciences of Dazhou City, Dazhou 635099, China

**Keywords:** *Tricholoma sinoportentosum*, *Termitomyces albuminosus*, polysaccharide, structure identification, immune activity

## Abstract

Polysaccharides are important active ingredients of living organisms. In this study, two new polysaccharides, *Tricholoma sinoportentosum* polysaccharide (TS-P) and *Termitomyces albuminosus* (TA-P), were extracted and purified using anion exchange column chromatography. The structure of each polysaccharide was identified by HPGPC, FT-IR, HPLC, GC-MS and NMR, and the biological activities were also investigated. The results of the structure identification showed that TS-P was composed of arabinose, mannose, glucose and galactose at a ratio of 1:1:3:2 and its main chain was composed of (1→4)-Arap residues, (1→4,6)-D-Manp residues and two (1→6)-Galp residues. The TA-P was composed of arabinose, glucose and galactose at a ratio of 2:4:8. Its main chain was composed of two (1→4)-β-L-Arap residues, one (1→4)-Glcp residues, three (1→2,6)-Galp residues and five (1→6)-Galp residues. The immunoassay showed that TS-P and TA-P could significantly promote the proliferation of T cells, B cells and RAW264.7 cells. The cell cycle results showed that for B cells and macrophages, TS-P and TA-P mainly affected the G0/G1 phases of the cell cycle; for T cells, TS-P affected G2/M phase, while TA-P mainly affected the G0/G1 phases. TS-P could significantly promote B cells to secrete IgA, IgG and IgD (*p* < 0.01), while TA-P could significantly promote the secretion of IgA and IgG (*p* < 0.01). The chemical structure and biological activity of TS-P and TA-P were first studied and compared to lay a theoretical foundation for the application of fungal polysaccharide.

## 1. Introduction

Currently, chemotherapy is still the main treatment for malignant tumors. Medicine resistance and toxic side effects generated during long-term chemotherapy are the main reasons for chemotherapy failure [1] Therefore, searching for natural products with low toxicity, high efficiency and anti-tumor activity has become a research hotspot. Fungi contain a variety of chemical components, such as polysaccharides, polypeptides, polyphenols, alkaloids, carotenoids, minerals, vitamins, terpenoids, etc. [2,3,4,5], and fungi polysaccharides have become the subject of attention because of their good biological activity, especially their immunomodulatory and anti-tumor effects. As early as the 1960s, Chihara et al. reported on the anti-tumor properties of lentinan [6,7,8]. More and more polysaccharides have been found to have biological activities such as anti-tumor, regulating immunity, anti-inflammatory and anti-diabetes [9,10]. Further study on the structure and biological activity of polysaccharides can greatly promote the research and development of edible and medicinal polysaccharides.

*Tricholoma sinoportentosum* belongs to *Basidiomycota*, *Agaricales*, *Tricholomataceae*, *Tricholoma*, which is an ectomycorrhizal fungus commonly growing in forest land distributed in Sichuan, Liaoning, Gansu and Jilin of China and called “white buckwheat fungus” with plump flesh, sturdy stipe and a unique flavor. The fruiting body of *Tricholoma sinoportentosum* is edible and has certain anti-cancer and inhibitory effect on sarcoma (S180) and Ehrlich’s carcinoma (EC) of mice [11]. Barros et al. determined the chemical composition and nutritional value of *Tricholoma sinoportentosum* from Northeast Portugal by HPLC. The results showed that mannitol and trehalose were the most abundant monosaccharide [12]. Iwona et al. found that β-Dextran was the most abundant content of the fruiting body of wild *Tricholoma sinoportentosum* from Poland, which had anti-cancer, anti-oxidation and immune regulation activities [13].

*Termitomyces albuminosus,* also known as termite fungus or chicken fungus, belongs to *Basidiomycotina*, *Agaricales*, *Tricholomataceae*, *Termitomyces*, which grows in mixed forests or on hillside grasslands in summer and autumn, mainly in Sichuan, Guizhou, Yunnan and Guangxi of China. It is a kind of rare, wild, large fungus symbiotic with termites, and the ant nest is an ideal symbiotic ecological environment. It was recorded in Li Shizhen’s Compendium of Materia Medica as early as the Ming Dynasty. *Termitomyces albuminosus* has delicious taste and rich nutrition; contains a variety of active ingredients such as polysaccharides, cellulose, polyphenols, crude protein, crude fat, etc.; and has a variety of pharmacological effects [14,15]. The research shows that the molecular weight of *Termitomyces albuminosus* polysaccharide (MPT-W) is 1.30 × 10^5^ Da, composed of Xyl, Fuc, Man, Gal and Glc with a molar ratio of 0.29:8.67:37.90:36.00:16.60 [16]. Zhao et al. found that the naturally depolymerized extracellular polysaccharide (DEPS) of *Termitomyces albuminosus* has antioxidant activity in vitro. The ability of DEPS to scavenge DPPH free radicals, hydroxyl free radicals and superoxide free radicals increased with the increase in DEPS concentration, and when the concentration was 400 μg/mL, the scavenging activity of DEPS reached (92.6 ± 5.12)%, (62.0 ± 3.18)% and (67.21 ± 3.56)%, respectively [17]. In addition, two kinds of polysaccharide, intracellular polysaccharide (EIPS) and acidic intracellular polysaccharide (AIPS), were obtained from *Termitomyces albuminosus*, and the pharmacological effects of EIPS and AIPS on lipid metabolism and oxidative stress results showed that EIPS and AIPS could inhibit the formation of hyperlipidemia induced by high-fat emulsion [18]. Zhao et al. discovered the protective effect of the *Termitomyces albuminosus* polysaccharide (RPTA) on acute alcoholic liver injury in mice [19]. He et al. found that the *Termitomyces albuminosus* polysaccharide has the functions of antioxidation, lowering blood lipid, analgesia, anti-inflammatory and immune regulation [20]. At present, there is no report on the structure and activity of polysaccharides from *Tricholoma sinoportentosum* and *Termitomyces albuminosus* in Yajiang County, Sichuan Province, China.

Yajiang County, under the jurisdiction of Ganzi Tibetan Autonomous Prefecture in Sichuan Province, China, belongs to the sub-humid climate zone of the Qinghai Tibet Plateau, with a wide variety of wild large fungi in its territory. At present, there is no report on the structure and activity of polysaccharides from *Tricholoma sinoportentosum* and *Termitomyces albuminosus* in Yajiang County. In this study, two new water-soluble polysaccharides, TS-P and TA-P, were extracted, isolated and purified from the fruiting bodies of *Tricholoma sinoportentosum* and *Termitomyces albuminosus,* respectively, which were collected in Yajiang County, Sichuan Province, China. The chemical structure and biological activity of TS-P and TA-P were first studied and compared to lay a theoretical foundation for the application of fungal polysaccharide.

## 2. Materials and Methods

### 2.1. Extraction, Separation and Purification of Polysaccharide

*Tricholoma sinoportentosum* and *Termitomyces albuminosus* were collected from Yajiang County, Sichuan Province, China. A total 200 g of dry fruiting body of *Tricholoma sinoportentosum* and *Termitomyces albuminosus* was grounded into powder, boiled in hot water three times and the supernatant was concentrated. Three times the volume of ethanol absolute was added to precipitate crude polysaccharides. The crude polysaccharides were purified by DEAE cellulose (DEAE-52) column using distilled water and 0.05, 0.1, 0.2 and 0.3 mol/L NaCl as the elution phase, and then dialysis (7000 Da, Biosharp, Shanghai, China) and drying in a vacuum freeze-drying machine (ALPHA2-4LD plus, Christ, Berlin, Germany) to obtain *Tricholoma sinoportentosum* and *Termitomyces albuminosus* polysaccharide, which were named TS-P and TA-P, respectively.

### 2.2. Polysaccharide Molecular Weight Analysis

The polysaccharide molecular weight was analyzed using high-performance gel permeation chromatography (HPGPC) [21].

### 2.3. Fourier Transform Infrared (FT-IR) Spectroscopy Analysis

A Fourier transform infrared spectrometer (Nicolet 5700, Thermo Scientific, New York, NY, USA) was used to obtain the data of polysaccharide (5 mg) mixed with dry KBr [22].

### 2.4. Methylation Analysis, GC-MS and Nuclear Magnetic Resonance (NMR) Assay

The derivatized product was detected by GC-MS (Agilent 7890A, NY, USA) [23]. The NMR spectra were analyzed by the Varian Unity INOVA 400/45 (Varian Medical Systems, New York, NY, USA) [24].

### 2.5. Effects of Polysaccharide on T Cell, B Cell and RAW264.7 Cell Proliferation and Cell Cycle

All cell lines were purchased from the cell bank of the Typical Culture Preservation Committee of the Chinese Academy of Science (Shanghai, China). Proliferation effects of polysaccharide (final concentration 1.25, 2.5, 5, 10 μg/mL) on cells were analyzed via CCK-8 method [25]. The cell cycles were measured at 488 nm using BD Flow Cytometry according to the cell cycle kit operating instructions.

### 2.6. Effects of Polysaccharide on the Release of Cytokines in T Cells, B Cells and RAW264.7 Cells

The release of cytokines was detected by the ELISA kit (Wuhan BOSTER Biological Technology Co., Ltd., Wuhan, China).

### 2.7. Statistical Analysis

Data are indicated as the mean ± standard deviation (SD). The significant difference compared with the blank control group is indicated by *, *p* < 0.05 and **, *p* < 0.01.

## 3. Results and Discussion

### 3.1. Elution Curve of TS-P and TA-P

**Figure 1:**
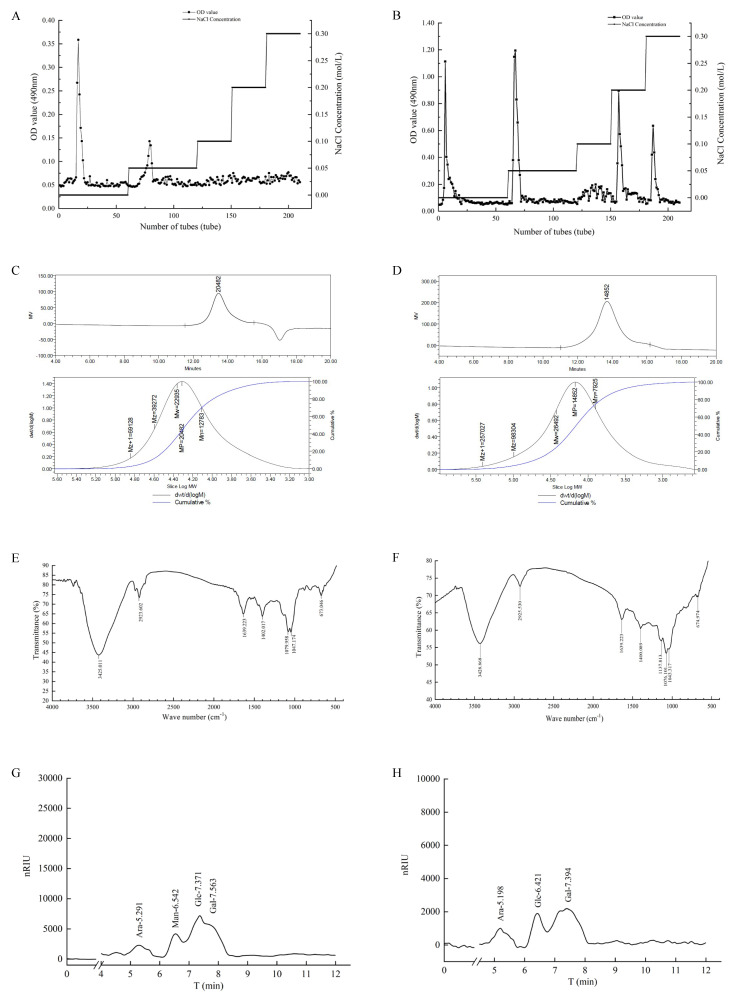
(**A**) Elution curve of TS-P by column chromatography. (**B**) Elution curve of TA-P by column chromatography. (**C**) Molecular weight of TS-P. (**D**) Molecular weight of TA-P. (**E**) Fourier transform infrared spectra of TS-P. (**F**) Fourier transform infrared spectra of TA-P. (**G**) Monosaccharide composition analysis of TS-P. (**H**) Monosaccharide composition analysis of TA-P.

Distilled water and 0.05, 0.1, 0.2 and 0.3 mol/L NaCl were used as eluents to elute polysaccharide from *Tricholoma sinoportentosum* and *Termitomyces albuminosus*, respectively. With the increase in NaCl concentration, there were two and four main elution peaks, respectively (Figure 1A,B). In this study, the first neutral polysaccharide eluates (the first peak in Figure 1A,B) in distilled water were collected as research objects as both the purity and content of these polysaccharides were suitable for subsequent research. The yields of TS-P and TA-P in the total fruiting body were about 2.52% and 3.19%, respectively.

### 3.2. Molecular Weight of TS-P and TA-P

The weight-average molecular weights (Mw) were 22,900 Da and 26,500 Da of polysaccharides TS-P and TA-P, respectively (Figure 1C). The peak molecular weight (Mp), the number-average molecular weight and the polydispersity are also shown in Figure 1C,D, which indicate that the molar mass distributions of these two polysaccharides are uniform.

### 3.3. FT-IR Analysis of TS-P and TA-P

The Fourier transform infrared spectrum of TS-P is shown in Figure 1E. A broad and strong absorption peak appeared at 3425 cm^−1^ and was designated as the stretching vibration peak of O-H; the absorption peaks at 2923 cm^−1^ and 1639 cm^−1^ were designated as the stretching vibration peaks of -CH_2_ and C=O, respectively; the absorption peak at 1402 cm^−1^ was designated as the bending vibration peak of the C-H plane; the stretching vibration peaks of C-O appeared at 1079 cm^−1^ and 1047.174 cm^−1^; the absorption peak at 673 cm^−1^ was specified as the bending vibration of -CH.

The results of the TA-P infrared spectrum are shown in Figure 1F. The wide and strong absorption peak at 3428 cm^−1^ was designated as the O-H stretching vibration peak; the peaks at 2925 cm^−1^, 1639 cm^−1^ and 1400 cm^−1^ were designated as the -CH_2_ stretching vibration peak, C=O stretching vibration peak and C-H bending vibration peak, respectively. The C-O stretching vibration peaks were at 1137 cm^−1^, 1076 cm^−1^ and 1043 cm^−1^. The signal at 674 cm^−1^ was the bending vibration peak of =C-H. In addition, there was no absorption peak near 1730 cm^−1^, indicating that TA-P does not contain uronic acid. The absorption peaks appearing at 1200–1000 cm^−1^ indicated that the monosaccharide rings both in TS-P and TA-P were pyran type.

### 3.4. Monosaccharide Composition Analysis of TS-P and TA-P

The HPLC results showed that the retention times of Rha, Xyl, Ara, Fru, Man, Glc and Gal were 4.51, 5.29, 5.83, 6.33, 6.58, 7.41 and 7.70 min, respectively. The HPLC results of TS-P after hydrolysis showed that the polysaccharide had four retention time peaks. Referring to the retention time of monosaccharide standard, it indicated that the peaks at the retention time of 5.29, 6.54, 7.37 and 7.56 min were the peaks of arabinose (Ara), mannose (Man), glucose (Glc) and galactose (Gal), respectively, and their peak area ratio was 1:1:3:2 (Figure 1G).

The results showed that the retention times of Rha, Xyl, Ara, Fru, Man, Glc and Gal were 4.21, 4.82, 5.29, 5.67, 5.83, 6.43 and 7.43 min, respectively. The HPLC results of TA-P after hydrolysis showed that the polysaccharide had three retention time peaks. The peaks at retention times of 5.20, 6.42 and 7.39 min were the peaks of arabinose (Ara), glucose (Glc) and galactose (Gal), respectively, and their peak area ratio was 1:2:4 (Figure 1H).

### 3.5. GC-MS Analysis of TS-P and TA-P

**Figure 2:**
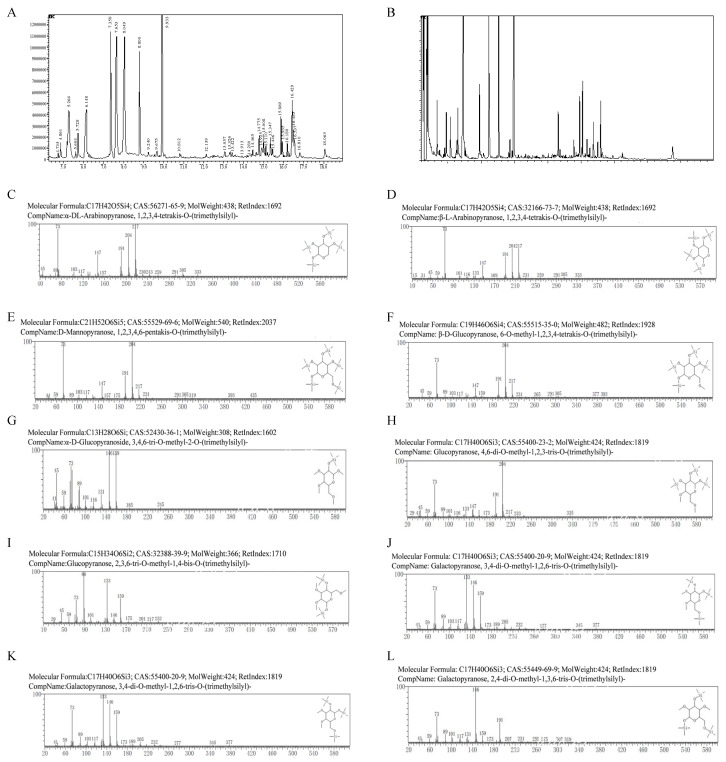
(**A**–**K**) The methylation analysis of TS-P. (**B**–**L**) The methylation analysis of TA-P.

The total ion spectrum of TS-P and TA-P was analyzed by GC-MS after methylation and silylation were carried. The GC-MS results of TS-P showed that β-arabinose residue was 1,2,3,4-tetrakis-O-trimethylsilyl-Arap; D-mannose residue was 1,2,3,4,6-pentakis-O-trimethylsilyl-Manp; and galactose residue was 3,4 di-O-methyl-1,2,6-tris-O-trimethylsilyl-Galp. Due to the large spatial steric hindrance of carbon at positions 2 and 3 on the monosaccharide ring, which was easy to form incomplete methylation, it is speculated that β-arabinose residues are 1,4-linked; D-mannose residues are 1,4,6-linked; and galactose residues are 1,6-linked. The α- D-glucose residue is 3,4,6-tri-O-methyl-2-O-trimethylsilyl-α-D-Glcp, indicating that there were 2-connected α-D-glucose residues. The other glucose residue is 2,3,6-tri-O-methyl-1,4-bis-O-trimethylsilyl-Glcp, indicating that there were 1,4-linked glucose residues in TS-P. These results indicated that TS-P mainly contained five types of monosaccharide residues, including (1→4)-β-arabinose residues, (1→4,6)-D-mannose residues, (1→6)-galactose residues, (1→4)-glucose residues and →2)-α-D-glucose residues (Figure 2A–K, Table 1).

The results showed that glucose residues were 4,6-di-O-methyl-1,2,3-tris-O-trimethylsilyl-Glcp and 6-O-methyl-1,2,3,4-tetrakis-O-trimethylsilyl-β-D-Glcp, and β-L-arabinose residue was 1,2,3,4-tetrakis-O-trimethylsilyl-β-L-Arap. Due to the large spatial steric hindrance of carbon at positions 2- and 3- on the monosaccharide ring, which could easily form incomplete methylation, it is speculated that there were glucose residues linked at positions 1- and 1,4- on the monosaccharide ring in TA-P. The linkage mode of β-L-arabinose residues is 1,4-linkage. The galactose residue were 3,4-di-O- methyl-1,2,6-tris-O-trimethylsilyl-Galp and 2,4-di-O-methyl-1,3,6-tris-O-trimethylsilyl-Galp. Considering the large steric hindrance of 2,3 carbon on the monosaccharide ring, which leads to incomplete methylation, there are 1,6-linked galactose residues in TA-P. These results indicate TA-P mainly contains five types of monosaccharide residues, including (1→4)-β-L-arabinose residues, (1→4)-glucose residues, (1→2,6)-galactose residues, (1→6)-galactose residues and →1)-glucose residues (Figure 2B–L, Table 2).

### 3.6. ^1^H-NMR Analysis of TS-P and TA-P

**Figure 3:**
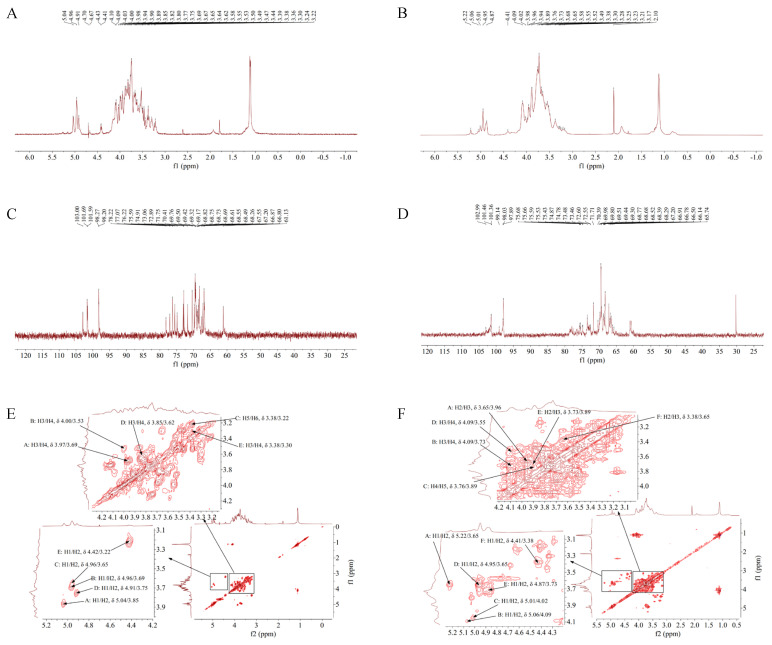
(**A**) ^1^H-NMR spectra of TS-P. (**B**) ^1^H-NMR spectra of TA-P. (**C**) ^13^C-NMR spectra of TS-P. (**D**) ^13^C-NMR spectra of TA-P. (**E**) ^1^H-^1^H COSY spectrum of TS-P. (**F**) ^1^H-^1^H COSY spectrum of TA-P.

NMR spectrum provides information for the structure of the compound such as integral curves, chemical shifts, peak shapes and coupling constants [26,27]. ^1^H-NMR spectra results of TS-P showed that TS-P had four anomeric proton signals, which were at δ 5.04, δ 4.96, δ 4.91 and δ 4.42, indicating that TS-P was composed of four monosaccharides in different chemical environments. Anomeric proton signals at δ 5.04, δ 4.91 and δ 4.42 belonged to →2)-α-D-Glcp (A), (1→4)-Glcp (D) and (1→4)-Arap (E), respectively. Anomeric proton signals at δ4.96 belonging to (1→4,6)-D-Manp (B) and (1→6)-Galp (C). The overlapping hydrogen signals between δ 3.22 and δ 4.41 were formed by H2–H6 in monosaccharides (Figure 3A, Table 3).

^1^H-NMR spectra results of TA-P showed that TA-P had six anomeric proton signals, which were at δ 5.22, δ 5.06, δ 5.01, δ 4.95, δ 4.87 and δ 4.41, indicating that TA-P was composed of six monosaccharides in different chemical environments. Anomeric proton signals at δ 5.22, δ 5.06, δ 5.01, δ 4.95, δ 4.87 and δ 4.41 belonged to (1→2,6)-Galp (A), (1→2,6)-Galp (B), (1→4)-Glcp (C), (1→6)-Galp (D), →1)-Glcp (E) and (1→4)-β-L-Arap (F), respectively. The overlapping hydrogen signals between δ 3.17 and δ 4.09 were formed by H2-H6 in monosaccharides. There was no signal peak after δ 5.40, indicating that there was pyranose and no furanose in both TS-P and TA-P (Figure 3B, Table 4).

### 3.7. ^13^C-NMR Analysis of TS-P and TA-P

^13^C-NMR spectra results of TS-P showed that TS-P had four anomeric carbon signals at δ 103.00, δ 102.00, δ 98.30 and δ 98.20, among which the signals at δ 103.00, δ 102.00 and δ 98.20 belonged to anomeric carbon signals of (1→4)-Arap (E), (1→4,6)-D-Manp (B) and (1→6)-Galp (C), respectively, and the signals at δ 98.27 belonged to →2)-α-D-Glcp (A) and (1→4)-Glcp (D). The signals between δ 61.10 and δ 78.20 were attributed to the carbon signal of C2-C6 in monosaccharide residues (Figure 3C, Table 3).

^13^C-NMR spectra results of TA-P showed that TA-P had six anomeric carbon signals at δ 103.00, δ 101.00, δ 101.00, δ 99.10, δ 98.00 and δ 97.90, which belonged to anomeric carbon signals of (1→4)-β-L-Arap (F), (1→2,6)-Galp (B), (1→6)-Galp (D), (1→2,6)-Galp (A), (1→4)-Glcp (C) and →1)-Glcp (E), respectively. The signals between δ 65.70 and δ 75.70 were attributed to the carbon signal of C2-C6 in monosaccharide residues. There was no signal peak in the range of δ 106–109, indicating that there was no furanose in both TS-P and TA-P, which is consistent with the results of ^1^H-NMR analysis (Figure 3D, Table 4).

### 3.8. ^1^H-^1^H COSY Analysis of TS-P and TA-P

The ^1^H-^1^H COSY spectrum can reflect the coupling relationship between adjacent hydrogen nuclei [28]. It solves the problem of partial proton signal overlap in polysaccharide and can obtain each proton signal of monosaccharide in polysaccharide. The ^1^H-^1^H COSY data of TS-P showed that signals A (δ 5.04/3.85), B (δ 4.96/3.69), C (δ 4.96/3.65), D (δ 4.91/3.75) and E (δ 4.42/3.22) were the resonance coupling signals between H-1 and H-2 of the →2)-α-D-Glcp (A), (1→4,6)-D-Manp (B), (1→6)-Galp (C), (1→4)-Glcp (D) and (1→4)-Arap (E) groups, respectively. The signals of H2-H6 of the A group were δ 3.85, δ 3.97, δ 3.69, δ 3.75 and δ 3.53, respectively, based on the coupling signal of adjacent ^1^H atoms. The ^1^H signals of B-E groups were also deduced (Figure 3E, Table 3).

The ^1^H-^1^H COSY data of TA-P showed that signals A (δ 5.22/3.6), B (δ 5.06/4.09), C (δ 5.01/4.02), D (δ 4.95/3.65), E (δ 4.87/3.73) and F (δ 4.41/3.38) were the resonance coupling signals between H-1 and H-2 of the (1→2,6)-Galp (A), (1→2,6)-Galp (B), (1→4)-Glcp (C), (1→6)-Galp (D), →1)-Glcp (E) and (1→4)-β-L-Arap (F) groups, respectively. The signals of H2-H6 of the A group were δ 3.65, δ 3.96, δ 3.55, δ 3.89 and δ 3.73, respectively (Figure 3F, Table 4).

### 3.9. HMQC Analysis of TS-P and TA-P

**Figure 4:**
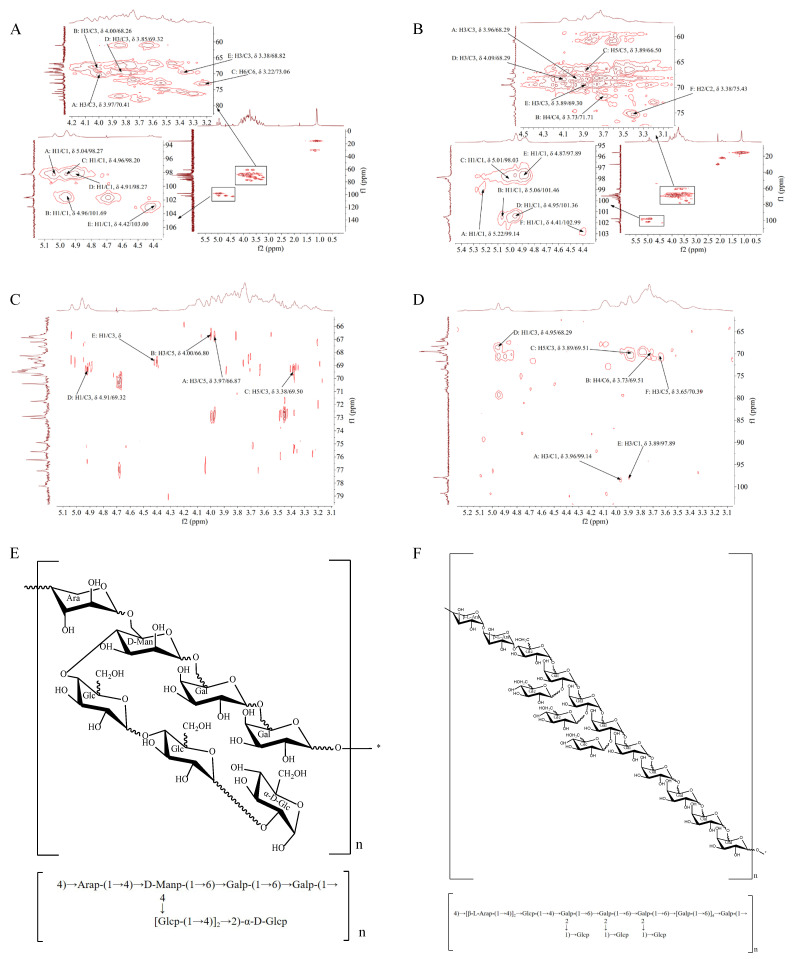
(**A**) HMQC spectrum of TS-P. (**B**) HMQC spectrum of TA-P. (**C**) HMBC spectrum of TS-P. (**D**) HMBC spectrum of TA-P. (**E**) Predicted chemical structure of TS-P. (**F**) Predicted chemical structure of TA-P.

HMQC provides directly connected ^1^H-^13^C relationship information [29]. The HMQC data of TS-P showed that signals A (δ 5.04/98.30), B (δ 4.96/101.00), C (δ 4.96/98.20), D (δ 4.91/98.30) and E (δ 4.42/103.00) were the resonance coupling signals of H-1 and C-1 of the →2)-α-D-Glcp (A), (1→4,6)-D-Manp (B), (1→6)-Galp (C), (1→4)-Glcp (D) and (1→4)-Arap (E) groups, respectively. The signals of C2-C6 in the →2)-α-D-Glcp (A) group were δ 69.50, δ 70.40, δ 69.80, δ 66.90 and δ 74.90, respectively. All the ^1^H signals of B-E groups were identified (Figure 4A, Table 3).

The HMQC data of TS-P showed that signals A (δ 5.22/99.10), B (δ 5.06/101.50), C (δ 5.01/98.00), D (δ 4.95/101.00), E (δ 4.87/97.90) and F (δ 4.41/103.00) were the resonance coupling signals of H-1 and C-1 of the (1→2,6)-Galp (A), (1→2,6)-Galp (B), (1→4)-Glcp (C), (1→6)-Galp (D), →1)-Glcp (E) and (1→4)-β-L-Arap (F) groups, respectively. The signals of C2-C6 in (1→2,6)-Galp (A) group were δ 68.40, δ 68.30, δ 69.40, δ 69.50 and δ 71.70, respectively. All the ^1^H signals of the B-F groups were also identified (Figure 4B, Table 4).

### 3.10. HMBC Analysis of TS-P and TA-P

HMBC data reflect the coupling relationship between ^1^H and remote ^13^C, which can provide the arrangement order and structural information of the molecular skeleton between monosaccharide residues [30]. In the HMBC data of TS-P (Figure 2I), the signal (δ 3.97/66.90) and signal (δ 4.00/66.80) were the resonance coupling signal between H-3 and C-5 of the →2)-α-D-Glcp (A) and (1→4,6)-D-Manp (B), respectively. The signal (δ 3.38/69.50) was the resonance coupling signal between H-5 and C-3 of the (1→6)-Galp (C). The signal (δ 4.91/69.30) and signal (δ 4.42/68.80) were the resonance coupling signal between H-1 and C-3 of the (1→4)-Glcp (D) and (1→4)-Arap (E), respectively (Figure 4C, Table 3).

The HMBC data of TA-P showed that the signal (δ 3.96/99.10) and (δ 3.89/97.90) were the resonance coupling signal H-3 and C-1 of the (1→2,6)-Galp (A) and →1)-Glcp (E), respectively, and the signal (δ 3.73/69.50), signal (δ 3.89/69.50), signal (δ 4.95/68.30) and signal (δ 3.65/70.40) were the resonance coupling signal between H-4 and C-6 of the (1→2,6)-Galp (B), H-5 and C-3 of the (1→4)-Glcp (C), H-1 and C-3 of the (1→6)-Galp (D), and H-3 and C-5 of the (1→4)-β-L-Arap (F) groups, respectively (Figure 4D, Table 4).

The structure of polysaccharide was identified by HPGPC, FT-IR, HPLC, GC-MS and NMR. The results showed that TS-P was composed of arabinose, mannose, glucose and galactose at a ratio of 1:1:3:2, and its main chain was composed of (1→4)-Arap residues, (1→4,6)-D-Manp residues and two (1→6)-Galp residues. Two (1→4)-Glcp residues were connected to (1→4,6)-D-Manp residues and one →2)-α-D-Glcp residue was connected to (1→4)-Glcp residues as the terminal group (Figure 4E).

The TA-P was composed of arabinose, glucose and galactose at a ratio of 2:4:8. Its main chain was composed of two (1→4)-β-L-Arap residues, one (1→4)-Galp residue, three (1→2,6)-Galp residues and five (1→6)-Galp residues. There were three branched chains, which were →1)-Glcp residues connected to (1→2,6)-Galp residues as the terminal group (Figure 4F).

Iwona et al. found that β-Dextran was the most abundant content of the fruiting body of wild *Tricholoma sinoportentosum* from Poland, which had anti-cancer, anti-oxidation and immune regulation activities [13]. Jia et al. reported that the molecular weight of *Termitomyces albuminosus* polysaccharide (MPT-W) is 1.30 × 10^5^ Da, composed of Xyl, Fuc, Man, Gal and Glc with a molar ratio of 0.29:8.67:37.90:36.00:16.60 [16]. However, currently, the primary structures of polysaccharide from *Tricholoma sinoportentosum* and *Termitomyces albuminosus* have not been identified. The molecular weight size is a necessary condition for polysaccharides to possess biological activity. The biological activity of polysaccharides usually requires their molecular weight to be within a certain range. The molecular weight of polysaccharides is too low to form advanced polymeric structures that generate activity; however, the larger the molecular weight and volume of polysaccharides, the less conducive they are to crossing multiple cell membrane barriers and entering the organism. Most of the polysaccharides with outstanding biological activity are connected by (1→3) or (1→4) glycosidic bonds. This configuration is conducive to the formation of a three strand spiral configuration, showing high biological activity. If the skeleton structure is mainly connected by (1→6) bonds or other bonds, the biological activity is low [31]. The degree of branch (DB), also known as degree of substitution (DS), also affects the activity of polysaccharides. Only when polysaccharides reach a certain degree of substitution can they have biological activity. Each polysaccharide has an optimal DB, enabling its biological activity to reach an ideal state [32,33]. In our study, the structure identification showed that TS-P (Mw 22,900 Da) was composed of arabinose, mannose, glucose and galactose at a ratio of 1:1:3:2 and its main chain was composed of (1→4)-Arap residues, (1→4,6)-D-Manp residues and two (1→6)-Galp residues. The TA-P (Mw 26,500 Da) was composed of arabinose, glucose and galactose at a ratio of 2:4:8. Its main chain was composed of two (1→4)-β-L-Arap residues, one (1→4)-Glcp residues, three (1→2,6)-Galp residues and five (1→6)-Galp residues. These two polysaccharides, TS-P and TA-P, are identified as new structures after SCIFinder query. Both polysaccharides conformed to the structural characteristics of molecular weight, main chain structure and branching degree, which could have potential biological activities.

### 3.11. Effect of TS-P and TA-P on B Cell Activity In Vitro

**Figure 5:**
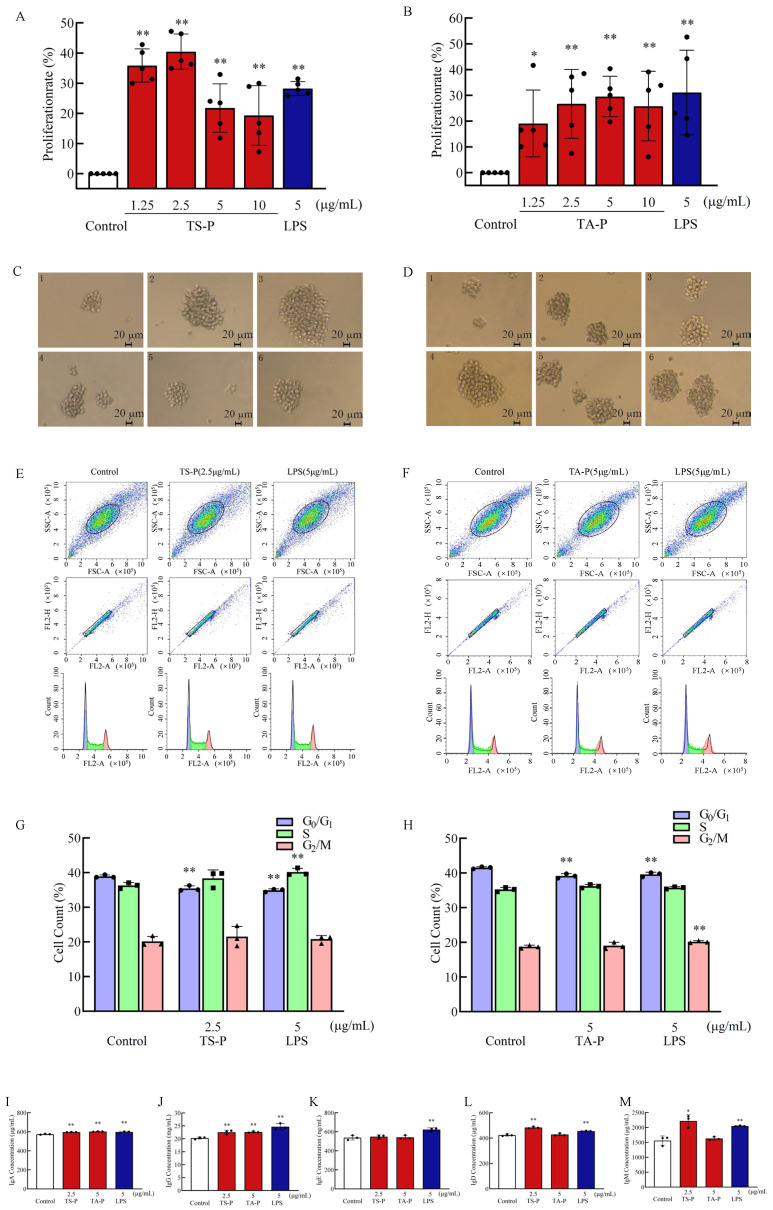
(**A**) Effect of TS-P on B cell proliferation. (**B**) Effect of TA-P on B cell proliferation. (**C**) Effect of TS-P on B cell morphology. (**D**) Effect of TA-P on B cell morphology. Notes: 1: control group; 2–5: 1.25, 2.5, 5 and 10 μg/mL of TA-P, respectively; 6: 5 μg/mL LPS; scale bars: 20 μm. (**E**) Effect of TS-P on B cell cycle. (**F**) Effect of TA-P on B cell cycle. (**G**) Statistic analysis of effect on the B cell cycle by TS-P. (**H**) Statistic analysis of effect on the B cell cycle by TA-P. (**I**–**M**) Effect of TS-P and TA-P on secretion of IgA, IgG, IgE, IgD and IgM by B cells, respectively. Notes: * indicates significant difference compared with the control (*p* < 0.05); ** indicates extremely significant difference compared with the control (*p* < 0.01).

B cells are pluripotent stem cells derived from bone marrow and play a huge role in humoral immunity. After recognizing and binding to external antigens through the surface receptors in B cells, extrinsic antigens activate the B cell signal pathway [34]. After a series of transformations, B cells differentiate into plasma cells and memory B cells. The plasma cells produce antibodies to remove pathogens to achieve an immune effect. The analysis of results indicated that the B cell proliferation effect was better than the LPS group (28.30%) when TS-P final concentrations were 1.25 and 2.5 μg/mL and the maximum proliferation rate was 40.47% (TS-P 2.5 μg/mL). The B cell proliferation effect was the best (29.60%) when the final concentration of TA-P was 5 μg/mL (Figure 5A,B). The observation results of cell morphology indicated that the number of B cells in the blank group is small, in bright spherical shape and in good condition. There was a certain dose relationship between the cell mass and TS-P or TA-P concentration, which was larger than that of the blank group at a final concentration of 1.25–10 μg/mL. The cells in the positive group also had no necrosis, grew well and were larger than those in the blank group (Figure 5C,D).

Cell cycle refers to the process that a continuously dividing cell goes through from the completion of one division to the completion of the next [35]. A cell cycle consists of G0/G1, S and G2/M phases. Based on the results of the effect of TS-P and TA-P on B cell proliferation, the optimal concentration 2.5 μg/mL of TS-P and 5 μg/mL of TA-P were selected for further study of the effect of TS-P and TA-P on the B cell cycle. The percentage of B cells in the G0/G1 phase decreased, while the percentage of B cells in S phase and G2/M phase increased under the stimulation of 2.5 μg/mL TS-P and 5 μg/mL TA-P. The number of B cells in the G0/G1 phase in the TS-P group significantly decreased from 38.90% to 35.40% compared with the blank group (*p* < 0.01). In addition, the G0/G1 phase and S phase of the positive group were significantly different from the blank group (*p* < 0.01). The percentage of G0/G1 phase cells decreased by 4.00%, while the percentage of S phase cells increased by 3.90%. The percentage of B cells in the G0/G1 phase decreased significantly (*p* < 0.01) by 2.37% and 1.90% under the stimulation of 5 μg/mL TA-P and 5 μg/mL LPS, respectively, Although the percentage of B cells in both the S phase and G2/M phase increased, only the percentage of B cells in the G2/M phase of the positive group had a very significant difference (*p* < 0.01) and the number of cells increased from 18.70% to 20.20%. The above results suggested that TS-P and TA-P both can eliminate stagnation of the cell cycle in the G0/G1 phase, thereby promoting the proliferation of B cells (Figure 5E–H).

Cytokines are hormone active protein molecules produced by immune cells after being stimulated by an antigen or mitogen [36,37]. The main function of B lymphocytes is to secrete immunoglobulin, which is the final stage of differentiation sequence occurring in different microenvironments in vivo. When the final concentration of TS-P is 2.5 μg/mL, it can significantly promote B cells to secrete IgA, IgG and IgD (*p* < 0.01), with secretion amounts of 597.00 μg/mL, 22.60 mg/mL and 484.00 μg/mL, respectively. It can significantly promote B cells to secrete IgM (*p* < 0.05), and the secretion amount was 2219.00 μg/mL. When the final concentration of TA-P is 5 μg/mL, it can significantly (*p* < 0.01) promote B cells to secrete IgA and IgG with secretion amounts of 603.00 μg/mL and 22.60 mg/mL, respectively. Compared with the blank group, the positive group can significantly promote B cells to secrete IgA, IgG, IgE, IgD and IgM (*p* < 0.01). It should be noted that the amount of IgD secreted by B cells stimulated by TS-P and the amount of IgA secreted by B cells stimulated by TA-P were higher than those in the positive group (Figure 5I–M).

### 3.12. Effect of TS-P and TA-P on T Cells Activity In Vitro

**Figure 6:**
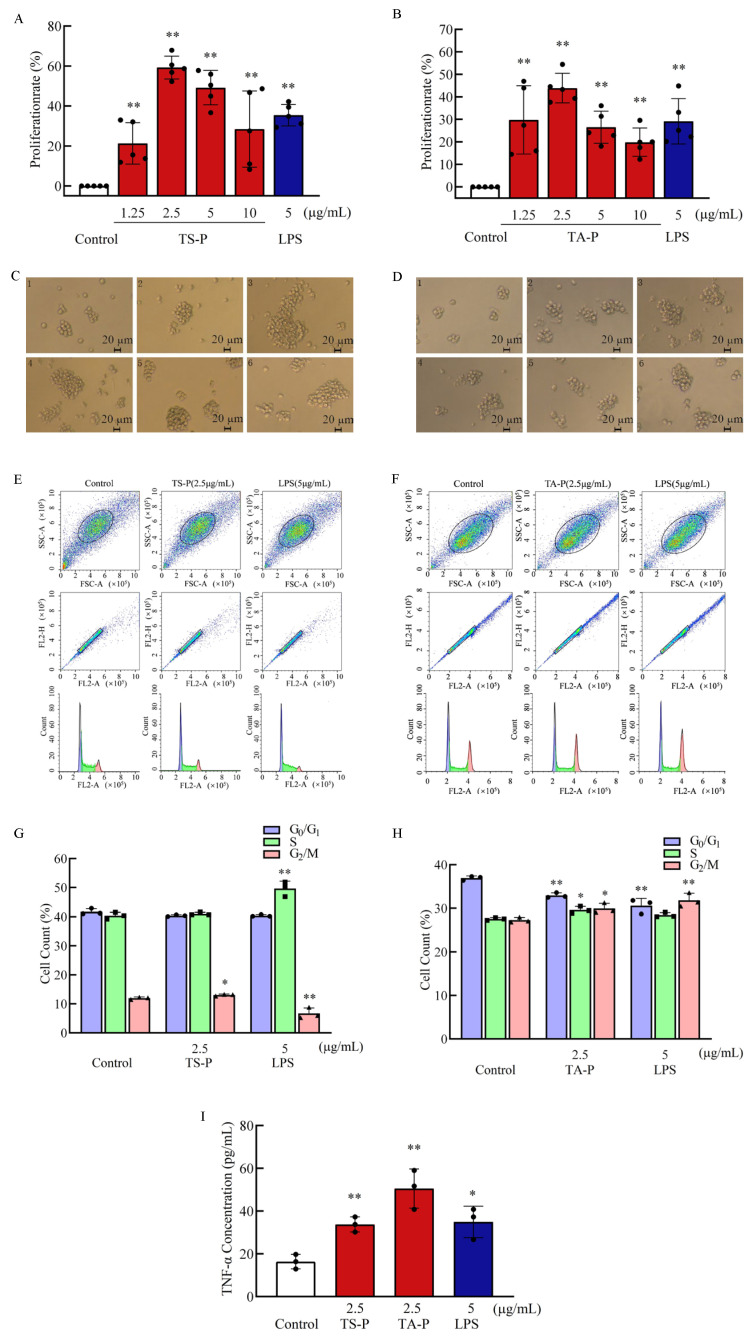
(**A**) Effect of TS-P on T cell proliferation. (**B**) Effect of TA-P on T cell proliferation. (**C**) Effect of TS-P on T cell morphology. (**D**) Effect of TA-P on T cell morphology. Notes: 1: control group; 2–5: 1.25, 2.5, 5 and 10 μg/mL of TA-P, respectively; 6: 5 μg/mL LPS; scale bars: 20 μm. (**E**) Effect of TS-P on T cell cycle. (**F**) Effect of TA-P on T cell cycle. (**G**) Statistic analysis of effect on the T cell cycle by TS-P. (**H**) Statistic analysis of effect on the T cell cycle by TA-P. (**I**) Effect of TS-P and TA-P on secretion of TNF-α by T cells, respectively. Notes: * indicates a significant difference compared with the control (*p* < 0.05); ** indicates an extremely significant difference compared with the control (*p* < 0.01).

T cells are differentiated from lymphoid stem cells in the thymus. They are the most numerous and complex type of cells in lymphocytes and participate in the cellular immunity of the body. Mature dendritic cells can recognize, process and present antigens to T cells, thus activating adaptive immune response [38]. The results showed that TS-P and TA-P could significantly promote the proliferation of T cells (*p* < 0.01) at the final concentrations of 1.25, 2.5, 5, 10 μg/mL compared with the blank group (Figure 6A,B). At the same time, the effect of the positive group on stimulating the proliferation of T cells also showed a significant difference (*p* < 0.01). The effect was better than that of the positive group (35.40%) when the final concentration of TS-P was 2.5 μg/mL or 5 μg/mL, and the proliferation effect of TS-P was the best at 2.5 μg/mL, the rate of which reached 59.30%. When the final concentration of TA-P was lower than 2.5 μg/mL, the proliferation rate was in direct proportion to it, and vice versa; when the final concentration of TA-P was 2.5 μg/mL, the maximum proliferation rate was 43.90%, which was higher than that in the positive group (29.20%). The observation results of cell morphology showed that the number of T cells stimulated by TS-P and TA-P increased and the cell clusters became larger compared with the blank group. To sum up, TS-P and TA-P can promote the proliferation of T cells in a certain concentration range, different concentrations of polysaccharides had different effects on the cell mass volume and cell number, both polysaccharides had a better proliferation effect on T cells at the final concentration of 2.5 μg/mL, and TS-P was superior to TA-P in stimulating T cell proliferation (Figure 6C,D).

Based on the results of the effect of TS-P and TA-P on T cells proliferation, the optimal concentration of 2.5 μg/mL of TS-P and TA-P was selected for further study on the effect of TS-P and TA-P on the T cell cycle (Figure 6E–H). The percentage of T cells in the S phase increased and the percentage of T cells in the G0/G1 phase decreased under 2.5 μg/mL TS-P compared with the blank group with no significant difference. At the same time, the percentage of T cells in the G0/G1 phase in the positive group decreased, and there were also no significant differences, but the percentage of T cells in the S phase increased by 9.30%, showing a very significant difference (*p* < 0.01); further, the percentage of T cells in the G2/M phase increased significantly (*p* < 0.05) from 12.0% to 13.1% in the TS-P group compared with the blank group. The percentage of T cells in the G0/G1 phase decreased significantly (*p* < 0.01) by 4.00% and 6.30%, respectively, under 2.5 μg/mL TA-P and 5 μg/mL LPS compared with the blank group, and the reduction in the positive group was greater than that in the TA-P group. The percentage of T cells in the S phase and G2/M phase of the TA-P and positive group increased, while the S phase and G2/M phase of the TA-P group showed a significant difference (*p* < 0.05) and the G2/M phase of the positive group showed an extremely significant difference (*p* < 0.01), increasing from 27.30% to 31.80%, compared with the blank group. These results suggested that the T cell cycle changed after being stimulated by TS-P and TA-P. The difference was that TS-P affected the cell cycle by affecting the G2/M phase—that is, by improving the ability of T cells to divide, while TA-P was mainly by affecting the G0/G1 phase.

The study on the effect of cytokines secreted by T cells is the verification of the functional changes of T cells after proliferation (Figure 6I). The secretory volumes of TNF-α were 33.70 pg/mL and 50.60 pg/mL when T cells were treated with TS-P (2.5 μg/mL) and TA-P (2.5 μg/mL), respectively, which both showed a very significant difference (*p* < 0.01). The positive group can significantly promote the secretion of TNF-α by T cells (*p* < 0.05) with the amount of secretion being 35.00 pg/mL, and it was not difficult to find that the TA-P group was better than the positive group. The above results indicated that TS-P and TA-P could not only promote the proliferation of T cells but also promote the secretion of TNF-α by T cells.

### 3.13. Effect of TS-P and TA-P on RAW264.7 Cells Activity In Vitro

**Figure 7:**
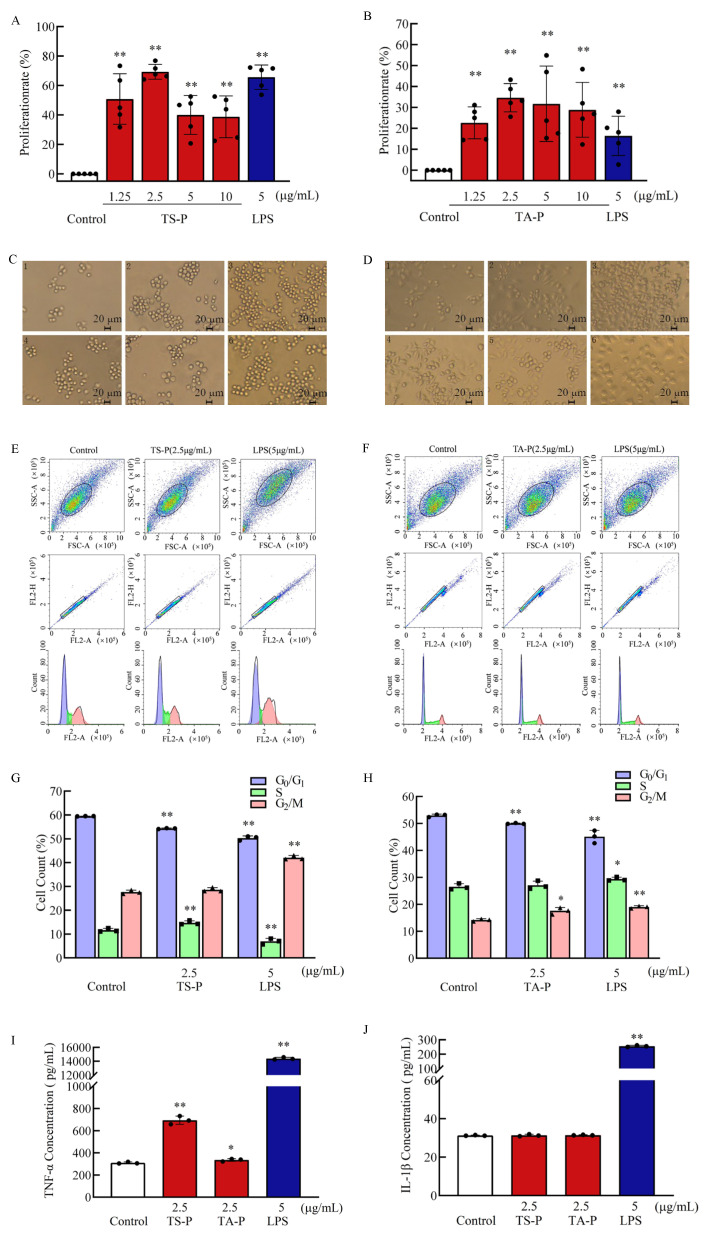
(**A**) Effect of TS-P on RAW264.7 cell proliferation. (**B**) Effect of TA-P on RAW264.7 cell proliferation. (**C**) Effect of TS-P on RAW264.7 cell morphology. (**D**) Effect of TA-P on RAW264.7 cell morphology. Notes: 1: control group; 2–5: 1.25, 2.5, 5 and 10 μg/mL of TA-P, respectively; 6: 5 μg/mL LPS; scale bars: 20 μm. (**E**) Effect of TS-P on RAW264.7 cell cycle. (**F**) Effect of TA-P on RAW264.7 cell cycle. (**G**) Statistic analysis of effect on the RAW264.7 cell cycle by TS-P. (**H**) Statistic analysis of effect on the RAW264.7 cell cycle by TA-P. (**I**,**J**) Effect of TS-P and TA-P on secretion of TNF-α and IL-1β by RAW264.7 cells, respectively. Notes: * indicates a significant difference compared with the control (*p* < 0.05); ** indicates an extremely significant difference compared with the control (*p* < 0.01).

Macrophages are mononuclear phagocytes involved in immune response, inflammation and many homeostasis processes [39]. The analysis of results showed that TS-P and TA-P can significantly promote the proliferation of RAW264.7 cells (*p* < 0.01) at the final concentrations of 1.25, 2.5, 5 and 10 μg/mL compared with the blank group (Figure 7A,B). The cell proliferation rate reached 69.30% at the final concentration of 2.5 μg/mL TS-P, which was equivalent to the positive group (65.60%). The maximum proliferation rate was 34.60% when the final concentration of TA-P was 2.5 μg/mL, which was higher than that of the positive group (16.40%). The observation results of cell morphology showed that the RAW264.7 cells in the blank group are round, almost without pseudopodia, and grow on the wall. With the increase in the final concentrations of TS-P and TA-P, the number of cells increased and some cells showed pseudopodia (Figure 7C,D).

Based on the results of the cell proliferation effects, the optimal concentration 2.5 μg/mL of TS-P and 2.5 μg/mL of TA-P were selected for further study on the effect of TS-P and TA-P on the RAW264.7 cell cycle (Figure 7E–H). The percentage of RAW264.7 cells in the G0/G1 phase of TS-P and the positive group were significantly reduced by 5.20% and 9.20%, respectively, compared with the blank group. The percentage of RAW264.7 cells in S phase in the TS-P group increased extremely significantly by 3.10% (*p* < 0.01). The percentage of RAW264.7 cells in the G2/M phase in TS-P and the positive group increased by 1.00% and 14.50% respectively; however, only the G2/M phase of the positive group had statistical significance (*p* < 0.01). The percentage of RAW264.7 cells in the G0/G1 phase of TA-P and positive group decreased, while the percentage of cells in the S phase and G2/M phase increased. The G0/G1 phase in TA-P group was significantly different from the blank group (*p* < 0.01) and the number of cells decreased from 52.97% to 50.00%. The percentage of cells of the positive group in the G0/G1 phase decreased by 7.87%, and the percentage of cells in the S phase and G2/M phase increased by 2.84% and 4.80%, respectively. These results show that TS-P and TA-P can affect the macrophage cycle by reducing the preparation time of G0/G1 to promote the proliferation of macrophages.

The secretion amounts of TNF-α under 2.5 μg/mL TS-P, 2.5 μg/mL TA-P and 5 μg/mL LPS were 694.00 pg/mL (*p* < 0.01), 336.02 pg/mL (*p* < 0.05) and 14,392.00 pg/mL (*p* < 0.01), respectively, and the secretion amounts of IL-1β were 31.40 pg/mL, 31.50 pg/mL and 256.00 pg/mL (*p* < 0.01), respectively. TS-P and TA-P could promote the secretion of TNF-α by macrophages with lower secretion amounts than the positive group but could not promote the secretion of IL-1β (Figure 7I,J).

As early as the 1960s, Chihara et al. reported the anti-tumor properties of shiitake mushroom polysaccharides [6]. More and more rare, edible and medicinal fungal polysaccharides in China have been discovered [40,41]. Zhou et al. found that *Grifola frondosa* polysaccharides could significantly promote macrophage production of NO and secretion of cytokines (TNF)-α, IL-1β and IL-δ [42]. Ding et al. reported that the *Gomphus clavatus* Gray polysaccharide could induce the apoptosis of HepG-2 cells and affect the mRNA expression of various housekeeping genes in the HepG-2 cells [43]. In our study, the two polysaccharides with novel structures could lead to immune cell proliferation by mainly affecting the G0/G1 phase of the cell cycle, and simultaneously promote the secretion of IgA, IgG and TNF-α by immune cells. Polysaccharide TS-P, which had more branching structures and lower molecular weight, displayed better proliferation effects on three types of cells than TA-P.

## 4. Conclusions

In this study, *Tricholoma sinoportentosum* polysaccharide (TS-P) and *Termitomyces albuminosus* polysaccharide (TA-P) were extracted and purified by hot water extraction, ethanol precipitation and DEAE-52 cellulose column chromatography. The molecular weight of *Tricholoma sinoportentosum* polysaccharide (TS-P) was 22,900 Da and that of *Termitomyces albuminosus* (TA-P) was 26,500 Da. The results of structure identification by GC-MS, HPGPC, FT-IR and NMR showed that TS-P was composed of arabinose, mannose, glucose and galactose at a ratio of 1:1:3:2, and its main chain was composed of (1→4)-Arap residues, (1→4,6)-D-Manp residues and two (1→6)-Galp residues. Two (1→4)-Glcp residues were connected to (1→4,6)-D-Manp residues and one →2)-α-D-Glcp residue was connected to (1→4)-Glcp residues as the terminal group. The TA-P was composed of arabinose, glucose and galactose at a ratio of 2:4:8. Its main chain was composed of two (1→4)-β-L-Arap residues, one (1→4)-Glcp residues, three (1→2,6)-Galp residues and five (1→6)-Galp residues. There were three branched chains, which were →1)-Glcp residues connected to (1→2,6)-Galp residues as the terminal group.

The immunoassay showed that TS-P and TA-P could significantly promote the proliferation of B cells, T cells and RAW264.7 cells. The cell proliferation activity of TS-P was better than that of TA-P. The optimal concentration of TS-P to stimulate the proliferation of the three kinds of cells was 2.5 μg/mL, with proliferation rates of 40.50%, 59.30% and 69.00%, respectively. The optimal concentrations of TA-P to stimulate the proliferation of the three kinds of cells are 5, 2.5 and 2.5 μg/mL, with proliferation rates of 29.60%, 43.90% and 34.60%, respectively. The cell cycle results showed that for B cells and macrophages, TS-P and TA-P mainly affected the G0/G1 phase of the cell cycle; for T cells, TS-P affected the G2/M phase and TA-P mainly affected the G0/G1 phase. TS-P can significantly promote B cells to secrete IgA, IgG and IgD (*p* < 0.01), while TA-P can significantly promote the secretion of IgA and IgG (*p* < 0.01). The amount of IgD secreted by B cells stimulated by TS-P and the amount of IgA secreted by B cells stimulated by TA-P were both higher than those in the positive group. Both TS-P and TA-P could significantly promote the secretion of TNF-α by T cells (*p* < 0.01) but only the TA-P group was significantly better than the positive group. Both TS-P and TA-P can promote the secretion of TNF-α by macrophages; the difference between the TS-P group and blank group was very significant (*p* < 0.01), the difference between the TA-P group and blank group was significant (*p* < 0.05), and both effects were lower than that of the positive group. Neither of them could promote the secretion of IL-1β by macrophages. The polysaccharide TS-P, which had more branching structures and smaller molecular weight, displayed better proliferation effects on three types of cells than TA-P.

At present, most of the research on edible and medicinal fungi is only at the early stages, but fungi polysaccharides have good biological activity and great application prospects in food and industrial development, which is worthy of further extensive research. Comparative studies on the structure and biological activities of the two new polysaccharides from *Tricholoma sinoportentosum* (TS-P) and *Termitomyces albuminosus* (TA-P) could provide certain scientific bases for the in-depth study of polysaccharides.

## Figures and Tables

**Table 1:** Analysis of methylation results of *Tricholoma sinoportentosum* polysaccharide (TS-P).

Methylated Product	Linkage	Unit (m/z)
1,2,3,4-tetrakis-O-trimethylsilyl-β-DL-Arap	1,4-	45 59 73 103 117 147 169 191 204 217 230 243 259 291 305 333
1,2,3,4,6-pentakis-O-trimethylsilyl-D-Manp	1,4,6-	44 59 73 89 103 117 147 157 175 191 204 217 231 291 305 319 393 435
3,4,6-tri-O-methyl-2-O-trimethylsilyl-α-D-Glcp	2-	41 45 59 73 89 101 116 131 146 159 185 245
2,3,6-tri-O-methyl-1,4-bis-O-trimethylsilyl-Glcp	1,4-	29 45 59 73 88 101 133 146 159 175 201 217 232
3,4-di-O-methyl-1,2,6-tris-O-trimethylsilyl-Galp	1,6-	41 59 73 89 103 117 133 146 159 173 189 205 232 277 345 377

**Table 2:** Analysis of methylation results of *Termitomyces albuminosus* polysaccharide (TA-P).

Methylated Product	Linkage	Unit (m/z)
1,2,3,4-tetrakis-O-trimethylsilyl-β-L-Arap	1,4-	15 31 45 59 73 101 116 133 147 169 191 204 217 231 259 291 305 333
6-O-methyl-1,2,3,4-tetrakis-O-trimethylsilyl-β-D-Glcp	1,4-	44 59 73 89 103 117 147 159 191 204 217 231 265 291 305 377 393
4,6-di-O-methyl-1,2,3-tris-O-trimethylsilyl-Glcp	1-	29 41 45 59 73 89 101 116 133 147 173 191 204 217 233 335
3,4-di-O-methyl-1,2,6-tris-O-trimethylsilyl-Galp	1,2,6-	41 59 73 89 103 117 133 146 159 173 189 205 232 277 345 377
2,4-di-O-methyl-1,3,6-tris-O-trimethylsilyl-Galp	1,6-	41 59 73 89 101 117 131 146 159 173 191 207 231 259 275 303 319

**Table 3:** Chemical shifts of H and C atoms of *Tricholoma sinoportentosum* polysaccharide (TS-P).

Glycosyl Residues	Chemical Shifts (ppm)					
	H1/C1	H2/C2	H3/C3	H4/C4	H5/C5	H6/C6
→2)-α-D-Glcp (A)	5.04/98.27	3.85/69.50	3.97/70.41	3.69/69.76	3.75/66.87	3.53/74.91
(1→4,6)-D-Manp (B)	4.96/101.69	3.69/71.75	4.00/68.26	3.53/74.91	3.85/66.80	3.65/68.26
(1→6)-Galp (C)	4.96/98.20	3.65/68.49	3.82/69.50	3.30/76.22	3.38/68.82	3.22/73.06
(1→4)-Glcp (D)	4.91/98.27	3.75/69.76	3.85/69.32	3.62/68.49	3.75/69.42	3.55/74.91
(1→4)-Arap (E)	4.42/103.00	3.22/72.89	3.38/68.82	3.30/69.50	3.65/69.17	--

**Table 4:** Chemical shifts of H and C atoms of *Termitomyces albuminosus* polysaccharide (TA-P).

Glycosyl Residues	Chemical Shifts (ppm)					
	H1/C1	H2/C2	H3/C3	H4/C4	H5/C5	H6/C6
(1→2,6)-Galp (A)	5.22/99.14	3.65/68.39	3.96/68.29	3.55/69.44	3.89/69.51	3.73/71.71
(1→2,6)-Galp (B)	5.06/101.46	4.09/69.44	4.09/68.39	3.73/71.71	3.89/66.50	3.52/69.51
(1→4)-Glcp (C)	5.01/98.03	4.02/68.77	4.02/69.51	3.76/69.30	3.89/66.50	3.65/68.29
(1→6)-Galp (D)	4.95/101.36	3.65/68.39	4.09/68.29	3.55/69.51	3.94/66.14	3.49/66.78
→1)-Glcp (E)	4.87/97.89	3.73/69.44	3.89/69.30	3.58/68.29	3.73/69.44	3.49/67.20
(1→4)-β-L-Arap (F)	4.41/102.99	3.38/75.43	3.65/73.48	3.25/69.51	3.49/70.39	--

## Data Availability

The data presented in this study are available on request from the corresponding author.
